# Reactive oxygen species-targeted therapeutic interventions for atrial fibrillation

**DOI:** 10.3389/fphys.2012.00311

**Published:** 2012-08-06

**Authors:** Ali A. Sovari, Samuel C. Dudley

**Affiliations:** Section of Cardiology, Center for Cardiovascular Research, University of Illinois at ChicagoChicago, IL, USA

**Keywords:** antioxidants, atrial fibrillation, mitochondria, NADPH oxidase, nitric oxide synthase, therapy

## Abstract

Atrial fibrillation (AF) is the most common arrhythmia that requires medical attention, and its incidence is increasing. Current ion channel blockade therapies and catheter ablation have significant limitations in treatment of AF, mainly because they do not address the underlying pathophysiology of the disease. Oxidative stress has been implicated as a major underlying pathology that promotes AF; however, conventional antioxidants have not shown impressive therapeutic effects. A more careful design of antioxidant therapies and better selection of patients likely are required to treat effectively AF with antioxidant agents. Current evidence suggest inhibition of prominent cardiac sources of reactive oxygen species (ROS) such as nicotinamide adenine dinucleotide phosphate (NADPH) oxidase and targeting subcellular compartments with the highest levels of ROS may prove to be effective therapies for AF. Increased serum markers of oxidative stress may be an important guide in selecting the AF patients who will most likely respond to antioxidant therapy.

## Introduction

Atrial fibrillation (AF), which affects approximately 2.5 million individuals in the United States, is the most common arrhythmia that requires medical treatment. The incidence of AF is increasing with the increase in the elderly population. The incidence of AF increases with age from less than 0.5 per 1000 person-years in individuals younger than 50 years to approximately 10 per 1000 person-years in those older than 70 years (Krahn et al., 1995). Miyasaka and colleagues estimated that 15.9 million people in the United States will have AF by 2050 (Miyasaka et al., 2006). The most serious adverse effects of AF are increased risk of stroke and peripheral embolization caused by thrombus formation mainly in the left atrial appendage (LAA). The incidence of stroke after a diagnosis of AF is 1–5% annually, depending on the patient's age and the presence of other risk factors.

The pathophysiology of AF is not completely understood. Reentrant circuits and ectopic activities particularly around the muscular sleeves of the pulmonary veins have been identified as potential electrophysiological mechanisms of AF (Jais et al., 1997; Van Wagoner, 2007; Iwasaki et al., 2011); however, these arrhythmias are probably the final representations of the underlying pathophysiological events in AF. Targeting the focal activities and reentry circuits without addressing the underlying pathologies, for example as catheter ablation therapy does, is likely to have constrained success because of this. Current antiarrhythmic drugs that usually block one or a few ion channels in cardiomyocytes are not highly effective in the treatment of AF, and they have shown serious adverse effects (Gjesdal, 2009). A better understanding of the pathophysiologic events upstream to reentry, focal activity and ionic current abnormalities are required to identify effective therapeutic targets.

Excess reactive oxygen species (ROS) have been implicated in pathogenesis of AF by affecting ion channels and propagation of the action potential. Nevertheless, oxidative stress biology is complex, and general radical scavengers have not shown impressive therapeutic effects in clinical trials (Sesso et al., 2008; Van Wagoner, 2008). This review will consider this paradox in further detail.

## Limitations of current therapies

Several limitations can be mentioned for the current available therapies for AF. The underlying reason for most of these shortcomings lies in the fact that the current therapies do not address the underlying pathophysiology of AF.

### Limitations of ion channel blockade

Current antiarrhythmic medications usually target one or a few ion channels and almost always block these channels. Table 1 summarizes the most common adverse effects of the antiarrhythmic drugs that are commonly used in the management of AF, and Table 2 provides a summary of most important clinical studies on proarrhythmic effects of antiarrhythmic drugs. Proarrhythmia is an adverse effect of all current antiarrhythmic agents, which suggests that proarrhythmia is an adverse effect of the current pharmacological approach rather than a side effect of a few of those drugs. The Cardiac Arrhythmia Suppression Trial (CAST) was a landmark clinical trial in which class IC antiarrhythmic agents were used to suppress premature ventricular contractions (PVCs) after myocardial infarction (MI) to reduce the risk of ventricular arrhythmias (CAST Investigators, 1989). The study rationale was that PVCs are associated with a higher rate of sudden arrhythmic death, and antiarrhythmic agents effectively suppress PVCs. Nevertheless, treatment with an antiarrhythmic drug in the CAST trial was associated with a higher cardiovascular mortality rate. The treatment strategy in CAST involved simply suppressing focal activity and blocking the sodium channel. Later, it was found that sodium channels are down-regulated in patients with heart failure (Santana et al., 2005), and therefore further blockade of those channels may promote arrhythmia.

**Table 1:** **The most common adverse effects of frequently used antiarrhythmic drugs in the management of atrial fibrillation**.

**Class**	**Drug**	Adverse effects^*^
IA	Quinidine	Nausea, vomiting, diarrhea, abdominal pain Tinnitus, hearing and visual disturbances, altered mental status Thrombocytopenia, hemolytic anemia, anaphylaxis Hypotension, QRS prolongation, syncope, torsades de pointes, QT prolongation
	Procainamide	Rash, myalgia, vasculitis Fever, agranulocytosis Drug-induced lupus Hypotension, QT prolongation, torsades de pointes, bradyarrhythmia
	Disopyramide	Urinary retention, constipation, glaucoma, xerostomia Negative inotropy QT prolongation, torsades de points
IB	Mexiletine	Tremor, anxiety, dysarthria, dizziness, diplopia, nystagmus Nausea, vomiting, gastrointestinal disturbance Hypotension, bradyarrhythmia
IC	Flecainide	Negative inotropy, bradyarrhythmia Decreases pacing threshold Altered mental status, irritability
	Propafenone	Dizziness, blurred vision Bronchospasm Bradyarrhythmia, heart failure exacerbation Decreases pacing threshold
II	Beta Blockers	Hypotension, bradyarrhythmia, heart failure exacerbation Bronchospasm Depression Sexual dysfunction
III	Amiodarone	Pulmonary fibrosis Abnormal liver function tests Abnormal thyroid function Bradyarrhythmia, heart failure exacerbation Tremor Photosensitivity Corneal deposits
	Dronedarone	Nausea, vomiting, diarrhea, and gastrointestinal disturbance Asthenic condition Bradycardia Skin rash Liver injury Increase cardiovascular mortality in patients with NYHA class IV or recent decompensated heart failure Increase risk of cardiovascular mortality, development of heart failure and stroke in permanent atrial fibrillation QT prolongation Hypokalemia and hypomagnesaemia with potassium-depleting diuretics
	Sotalol	Bradyarrhythmia, torsades de pointes
IV	Calcium Channel Blocker (Verapamil)	Hypotension, bradyarrhythmia

* A common adverse effect of all the above antiarrhythmic medications is proarrhythmia.

**Table 2:** **Clinical studies on the proarrhythmia of antiarrhythmic drugs**.

**The Vaughn Williams class of antiarrhythmic**	**Clinical Studies on the proarrhythmic effects**
Class IA (Quinidine, procainamide, and disopyramide)	– A meta-analysis of six clinical studies showed that using quinidine for atrial fibrillation management is associated with more than 3 times higher mortality (2.9% vs. 0.8%, the quinidine-treated and no quinidine patients respectively, *p* < 0.05) (Coplen et al., 1990).
	– A meta-analysis of four clinical trials showed that quinidine was associated with significantly higher arrhythmia and sudden arrhythmic death than flecainide, mexiletine, and propafenone with 11 sudden cardiac deaths among 506 patients who were treated with quinidine (Morganroth and Goin, 1991).
Class IB (Lidocaine, tocainide, mexilitine, and diphenylhydantoin)	– A small study of patients with Wolff-Parkinson-White and atrial fibrillation suggested that lidocaine may increase pre-excitation and ventricular rate in atrial fibrillation (Akhtar et al., 1981).
Class IC (Flecainide, propafenone, and moricizine)	– The landmark study, the Cardiac Arrhythmia Suppression Trial, showed that total and cardiovascular mortality increases with the use of these drugs in patients after myocardial infarction despite suppression of premature ventricular beats (CAST Investigators, 1989).
	– The Cardiac Arrest Study Hamburg (CASH) showed that using propafenone in patients after a sudden cardiac arrest is associated with significantly higher mortality compared to using beta blocker or amiodarone (Siebels et al., 1993).
Class III (Amiodarone, sotalol, bretylium, dofetilide, azimilide, and ibutilide)	– Although these drugs and particularly amiodarone are effective in acute treatment of sudden cardiac death several large clinical trials have shown no survival benefit from using these drugs compared to placebo probably because of their proarrhythmic effect in long term use. The European Myocardial Infarct Amiodarone Trial (EMIAT) revealed that amiodarone in patients after myocardial infarction with left ventricular ejection fraction < 40% has no survival benefit compared to placebo (Julian et al., 1997). Survival Trial of Antiarrhythmic Therapy in Congestive Heart Failure, a double blind randomized clinical trial in the United States that studied 674 symptomatic heart failure patients with ejection fraction < 40% and at least 10 premature ventricular beats per hour did not show any survival benefit for amiodarone compared to placebo (Singh et al., 1995). A Canadian study similarly showed no benefit from amiodarone in prevention of sudden cardiac death (Cairns et al., 1997). Use of d-sotalol in patients with MI may be associated with increased mortality (Waldo et al., 1996).

Targeting ion channels as a therapeutic strategy carries the disadvantage of a narrow therapeutic index in which both low and high currents can cause arrhythmia. In addition, AF affects more than one ion current and blockade of one current may even potentiate the current imbalance toward arrhythmia.

### Limitations of catheter ablation

Catheter ablation uses tissue destruction to block the propagation of the focal activity or to disrupt reentrant circuits. Catheter ablation has achieved considerable success in treating certain types of arrhythmia. Catheter ablation is an anatomically fixed treatment that may be the best choice for patients with an anatomically fixed substrate. For example, a bypass tract between the atria and the ventricles can be treated effectively with catheter ablation. Nevertheless, AF is often a complex arrhythmia with widespread and dynamic substrates. Thus, a line of ablation that cuts the current reentrant circuit may not be an effective treatment for future AF, since the substrate of arrhythmia may change location over time. The necessity to continue anticoagulation for prevention of stroke after catheter ablation of AF suggests the lack of complete suppression of AF by this treatment. In addition, the ablation-generated fibrotic scar tissue may provide an arrhythmogenic substrate and the procedure is associated with some immediate and long term complications (Maan et al., 2011).

## ROS and their cardiac sources

### Reactive oxygen species

The term ROS refers to a class of low molecular weight molecules that are partially reduced derivatives of molecular oxygen. ROS are wide range of molecules that include the superoxide radical anion (O_2_^•−^); hydrogen peroxide (H_2_O_2_); the hydroxyl radical (OH^•^ + OH^−^); peroxynitrite (ONOO^−^), which is the product of the diffusion-controlled reaction between ^•^NO and O_2_^•−^; and the derived radicals ^•^NO_2_ and CO_3_^•−^. Low levels of ROS are necessary to mediate physiologic responses and to maintain homeostasis through the regulation of signal transduction events. Nevertheless, when cellular levels of ROS exceed the cell's ability to reduce excess free radicals, oxidative stress develops. The physiological concentration of ROS molecules may vary under different conditions and in different cellular compartments. In addition, methods of ROS measurement have certain limitations (Tarpey and Fridovich, 2001). It is generally thought that the intracellular concentration of superoxide rarely exceeds 1 nM (Brawn and Fridovich, 1980; Tarpey et al., 2004), and the normal physiological concentration of H_2_O_2_ is less than 15 μM (Tarpey and Fridovich, 2001; Kulagina and Michael, 2003; Liu et al., 2004; Tarpey et al., 2004). How much ROS increase in different pathological conditions may greatly vary; however, the known physiological range of ROS can provide a general guide to use relevant ROS concentrations and avoid extremely high levels of ROS in experimental studies.

Most ROS react with multiple biomolecules (proteins, deoxyribonucleic acid, ribonucleic acid, and lipids) and cause the loss of enzyme function, breaks in DNA strands, DNA mutations, lipid peroxidation, and cellular death. Protein cross-links, fragmentation, hydroxylation, nitration, halogenation, carboxylation, and reactive aldehyde formation are common outcomes of the interaction of proteins with various oxidants. Most of the ROS effects on proteins are irreversible and result in loss of function of those proteins, which eventually are degraded and removed by proteasomes (Levine, 2002). An aggressive ROS molecule such as hydroxyl radical can modify most amino acids (Halliwell et al., 1987); however, some amino acids such as cysteine, methionine, proline, arginine, tyrosine, and tryptophan have generally higher susceptibility to ROS modifications.

One important way in which ROS exert their effect is by modifying the thiol group of proteins (cysteine amino acid contains thiol groups; Chen et al., 2003) often times interfering with signal transduction cascades what leads to low pKa thiol phosphatase inhibition with consequent augmentation of kinase activity (Sommer et al., 2002; Connor et al., 2005). In addition to direct oxidation of thiol groups of proteins, ROS can oxidize low molecular weight biomolecules such as glutathione generating secondary oxidative products that then may react with protein thiols (Eaton, 2006). Methionine residues can be oxidized to methionine-S-sulfoxides and methionine-R-sulfoxides, which may be a reversible process; however, further oxidation of methionine residues to methionine-S-sulfone seems to be irreversible (Weissbach et al., 2002). An additional effect of ROS on amino acids is reactive aldehyde formation. It has been shown that ROS (and particularly HOCl) oxidize almost all amino acids commonly found in the plasma to a corresponding family of aldehydes in high yield (Hazen et al., 1998a,b). The reactive aldehydes have been shown to mediate the effect of ROS in cardiovascular disorders such as atherosclerosis and in diabetes (Uchida, 2000). Carbonylation of proline, lysine, threonine, and arginine is another important protein modification by ROS (Levine, 2002). In failing explanted human hearts, an increase in the carbonylation of actin and tropomyosin, and an increase in the dimerization and nitrosation of tropomyosin have been reported as evidence of the oxidative modifications of important cardiac proteins (Canton et al., 2011). Table 3 summarizes some of the important modifications of amino acids by oxidative stress.

**Table 3:** **Some important amino acid modifications by reactive oxygen/nitrogen species**.

**Modification**	**Reaction description**	**Most commonly affected amino acids**
Thiol modification (Barford, 2004)	It results in formation of sulfenic acids, intra- and intermolecular disulfides, cyclic sulfenamides, glutathionylation, sulfenyl-amide linkages, and *S*-nitrosation. Some of the reactions are reversible	Cysteine
Methionine oxidation (Stadtman et al., 2003)	Similar to cysteine, methionine has sulfur in its structure. Its oxidation by ROS results in formation of methionine sulfoxide. The reaction is reversible by methionine sulfoxide reductases. Further oxidation to methionine-S-sulfone may not be reversible	Methionine
Nitrosylation (Alvarez and Radi, 2003)	Addition of nitrosyl group to the protein. S-nitrosation refers to the reaction with cysteine and methionine	Cysteine, methionine, tyrosine, tryptophan, phenylalanine, histidine
Carbonylation (Wong et al., 2010)	Introducing the carbonyl group to the amino acid. May be reversible by a decarbonylation process. Carbonyl groups may form cross linkage with lysine residue of another protein. Detection of carbonylated proteins is an important method for detection of the ROS effect	Proline, arginine, lysine, threonine
Reactive aldehyde formation (Hazen et al., 1998a,b)	ROS (particularly HOCl) can virtually affect all amino acids to form reactive aldehydes. Generally irreversible	Most amino acids

ROS reactions can also lead to the formation of lipid hydroperoxides, which are oxygenated products of the primordial lipid radical. Lipid peroxidation is self-perpetuating and thus amplifies several-fold the initial damage of ROS-induced oxidation. The accumulation of reactive lipid peroxides and lipid-derived aldehydes also contributes to oxidant-mediated signaling and cell damage. DNA is a frequent target of ROS. The most common ROS-induced modifications to DNA include single-strand breaks. Double-strand breaks are potentially hazardous to cells and are repaired via two main pathways: homologous recombination and nonhomologous end-joining. In addition to strand breakage, hydroxylation, adduct formation, and the nitration of bases can damage the DNA. Repair mechanisms exist that are largely dependent on base excision, replacement, and relegation (Riis and Poulsen, 2005).

### Cardiac sources of ROS

**Figure 1:**
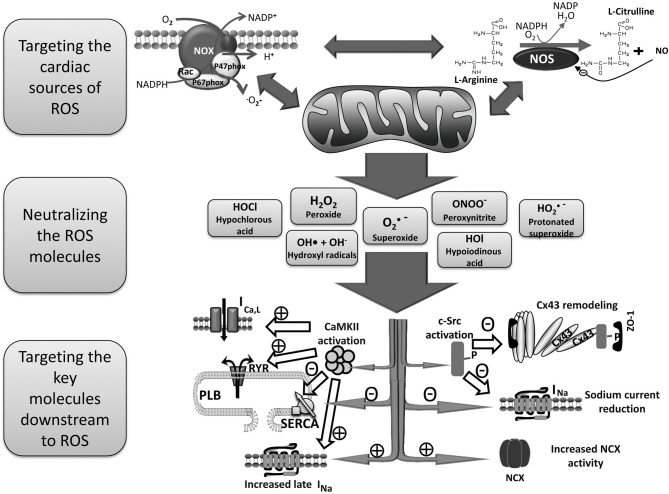
**Schema for the treatment of AF by reducing ROS.** Mitochondria, NADPH oxidase and uncoupled NOS are probably the most important cardiac sources of excess ROS. There are positive feedbacks among these sources in a way that activation of one results in increased activity and ROS production of the others. A variety of ROS molecules are produced as a result of activation of those sources of cardiac ROS which then oxidize proteins and lipids resulting in arrhythmia in several ways. Three main therapeutic strategies to prevent ROS induced arrhythmia are targeting the main cardiac sources of ROS, neutralizing ROS molecules, and searching for the key molecules that mediate the arrhythmogenic effects of ROS. AF, atrial fibrillation; CaMKII, Ca^2+^/calmodulin-dependent protein kinases II; CX43, connexin43; NADPH, nicotinamide adenine dinucleotide phosphate; NCX, Na^+^/Ca^2+^ exchanger; NOS, nitric oxide synthase; PLB, phospholamban; ROS, reactive oxygen species; RYR, ryanodine receptor, SERCA, sarco/endoplasmic reticulum Ca^2+^-ATPase.

Of the numerous cellular sources of ROS generation, mitochondria, the enzyme nicotinamide adenine dinucleotide phosphate (NADPH) oxidase, and uncoupled NOS are considered the major ROS production systems in the human heart. Those sources of cardiac ROS are interrelated and often activation of one results in activation of the others (Doughan et al., 2008; Zinkevich and Gutterman, 2011; Figure 1). For example, mitochondrial function differentially modulates NADPH oxidase expression and activity (Wosniak et al., 2009; Kuroda et al., 2010).

In mitochondria, when electrons flow from nicotinamide adenine dinucleotide (NADH) and flavin adenine dinucleotide to molecular oxygen via complex shuttle mechanisms, partially reduced semiquinone intermediates are produced and can react directly with O_2_ to produce O_2_^•−^. The coenzyme Q semiquinone is believed to be the major point of electron leakage in mitochondria.

The NAD(P)H oxidase is an enzyme that uses NAD(P)H to reduce molecular oxygen and produces large amounts of superoxide radicals. ROS are generated as a primary product of the NAD(P)H oxidase system, rather than a byproduct as they are in the mitochondrial system. Although NAD(P)H oxidase activity was first described in macrophages, a number of NAD(P)H oxidase isoforms (the Nox family) have been discovered in variety of nonphagocytic cells and Nox1, Nox2, and Nox4 have been detected in the heart. Nox activity is regulated in part by angiotensin II (Sorescu et al., 2002).

Uncoupled or dysfunctional nitric oxide synthases (NOSs) are other important sources of ROS. There are three major isoforms of NOS enzymes: inducible NOS (iNOS), endothelial NOS (eNOS), and neuronal NOS (nNOS). Their basic function is oxidizing the terminal guanidine nitrogen atom of L-arginine by using electrons from NADPH to produce NO., as shown below:

$$\begin{array}{c} \text{2L-arginine}+4{\text{O}}_{2}+\text{3NADPH}+{\text{3H}}^{+}=>\text{2L-citrulline}\\ +2{\text{NO}}^{•}+{\text{3NADP}}^{+}+{\text{4H}}_{2}\text{O} \end{array}$$

NO^•^ increases the cGMP level. It reversibly binds to and inhibits complex IV in mitochondria (Brown and Cooper, 1994), and it may nitrosate some thiols (Stamler and Hausladen, 1998). The oxidation of NO^•^ leads to the formation of ^•^NO_2_, which is also involved in redox signaling and direct tissue damage. NO^•^ signaling is diverse. One important interaction is the reaction of NO^•^ with superoxide, the product of which is peroxynitrite (ONOO^−^/ONOOH). The formation of ONOO^−^/ONOOH can directly or via the depletion of tetrahydrobiopterin (BH_4_) lead to NOS uncoupling, which further reduces NO^•^ production. Increased superoxide production has been shown to reduce NO^•^ signaling with detrimental effects on endothelial cell homeostasis (Freeman et al., 1995).

In addition to mitochondria, NAD(P)H oxidase, and NOS systems, there are other sources of ROS (e.g., peroxidases, xanthine oxidase, and aldehyde oxidase), the roles of which in cardiac oxidative stress are less well-documented than these three sources.

## ROS in atrial fibrillation

AF is associated with several clinical risk factors including some cardiac and respiratory disorders, aging, and surgeries. Table 4 shows the main clinical risk factors of AF. An elevated level of ROS has been linked with increasing age, heart failure, diabetes mellitus, coronary artery disease, obesity, and alcohol intoxication, all conditions associated with AF (Sovari and Dudley, 2010). In addition, multiple studies have shown an increase in markers of oxidative stress in humans with AF. The activity of myofibrillar creatine kinase (MM-CK), which is redox sensitive, can be used as an indirect marker of oxidative stress (Mihm et al., 2001). MM-CK activity is decreased, and immunodetectable 3-nitrotyrosine, a marker for the presence of peroxynitrite, is increased in right atrial appendage (RAA) of patients with AF compared to those in normal sinus rhythm (Mihm et al., 2001). Moreover, coronary artery bypass surgery, a procedure that is associated with AF in a considerable percentage of patients, is linked with an increase in oxidized glutathione and lipid peroxidation (De Vecchi et al., 1998). Derivatives of reactive oxidative metabolites (DROMs) and ratios of oxidized to reduced glutathione [E(h) GSH] and cysteine [E(h) CySH] that quantify oxidative stress in patients are increased in patients with AF (Neuman et al., 2007). The increase in the odds ratios for AF for an elevated E(h) GSH, E(h) CySH, or DROMs are 6.1 (95% CI, 1.3–28.3; *P* = 0.02), 13.6 (95% CI, 2.5–74.1; *P* = 0.01), and 15.9 (95% CI, 1.7–153.9; *P* = 0.02), respectively (Neuman et al., 2007).

**Table 4:** **Main clinical risk factors of atrial fibrillation and their association with oxidative stress**.

**Risk factors of atrial fibrillation**	**Reference**
**CARDIAC DISEASES**	
Hypertension	(De Champlain et al., 2004)
Coronary artery disease	(Vassalle et al., 2004; Madamanchi et al., 2005)
Cardiomyopathies and heart failure	(Ide et al., 2000; Sam et al., 2005)
Valvular diseases	(Liberman et al., 2008; Miller et al., 2008)
**PULMONARY DISEASES**	
Pulmonary embolism	(Ovechkin et al., 2007)
Chronic obstructive pulmonary disease	(Hattori et al., 1997)
Obstructive sleep apnea	(Yamauchi et al., 2005)
Pneumonia	(Duflo et al., 2002)
**SURGERIES**	
CABG and valve surgeries	(Milei et al., 2001)
Cardiac transplantation	(Kofler et al., 2008)
**OTHER DISEASES AND CONDITIONS**	
Aging	(Kregel and Zhang, 2007)
Hyperthyroidism	(Civelek et al., 2001)
Diabetes Mellitus and obesity	(Li et al., 2008)
Autonomic dysfunction	(Irigoyen et al., 2005)
Alcohol	(Cederbaum, 2001)

From Sovari, A. A. and Dudley, S. C. (2010). “Atrial Fibrillation and oxidative stress,” in Studies on Cardiovascular Disorders, Oxidative Stress in Applied Basic Research and Clinical Practice, 1st Edn., eds H. Sauer and A. Shah (Humana Press – Springer Science), 373–387; ISBN-13: 978-1607615996.

Kim and colleagues measured NAD(P)H-stimulated superoxide production in RAA, and plasma markers of lipid and protein oxidation (thiorbabituric acid-reactive substances, 8-isoprostane, and protein carbonyls) in 170 patients undergoing coronary artery bypass surgery. They found that NAD(P)H oxidase activity was the strongest independent predictor of postoperative AF (odds ratio 2.41; 95% confidence interval 1.71–3.40, *p* < 0.0001; Kim et al., 2008).

In addition, genetic studies have shown that the gene expression pattern of atrial tissue in patients with AF is associated with oxidative stress. AF in human has been shown to be associated with a significant reduction in the gene expression of antioxidant genes as well as a significant increase in the gene expression of five genes related to ROS, supporting a clear shift toward pro-oxidation state in AF (Kim et al., 2003). The gene expression of glutathione peroxidase-1 and heme oxygenase-2 are decreased while the gene expression of flavin containing monooxygenase-1, monoamine oxidase-B, uniquin specific protease-8, tyrosine-related protein-1, and tyrosine 3-monooxygenase are increased in atrial tissue of AF patients (Kim et al., 2003). For a more complete list of gene expression and protein level changes of pro-oxidant and antioxidant enzymes in AF patients please see Table 5.

**Table 5:** **Gene expression and protein level of pro-oxidants and antioxidants in the right atrial appendage of AF patients compared to patients in sinus rhythm**.

	**Gene expression**	**Protein level**
**PRO-OXIDANTS**		
Monoamine oxidase B (Kim et al., 2003)	⇑⇑⇑	
Flavin containing monooxygenase 1 (Kim et al., 2003)	⇑⇑⇑	
Tyrosinase-related protein 1 (Kim et al., 2003)	⇑⇑⇑	⇑
Tyrosine 3-monooxygenase (Kim et al., 2003)	⇑⇑	⇑
Ubiquitin specific protease 8 (Kim et al., 2003; Reilly et al., 2011b)	⇑⇑	
NAD(P)H oxidase (Kim et al., 2003; Reilly et al., 2011b)	⇑	⇑
Cytochrome P 450 (Kim et al., 2003)	⇑	
Xanthine oxidase (Kim et al., 2003)	⇑	
**ANTI-OXIDANTS**		
Peroxiredoxin 3 (Ohki et al., 2005)	⇓⇓⇓	
Glutathione peroxidase 1 (Kim et al., 2003)	⇓⇓⇓	
Heme oxygenase (decycling) 2 (Kim et al., 2003)	⇓	⇓
Glutaredoxin (thioltansferrase) (Kim et al., 2003)	⇓	
Glutathione reductase (Kim et al., 2003)	⇓	
Superoxide dismutase (Kim et al., 2003)	⇓	
Catalase (Kim et al., 2003)	⇓	

Recent studies on the effect of omega-3 fatty acids on ROS production have produced conflicting results (Kowey et al., 2010; Liu et al., 2011), and it was shown that some forms of polyunsaturated fatty acids may even increase oxidative stress (Kimura et al., 2012), which may provide an explanation for some of the associated proarrhythmic effects that have been seen with omega-3 fatty acids (Billman et al., 2011).

## Mechanisms of ROS induced arrhythmia

The data supporting the association of excess ROS with human AF is in the form of increased oxidized glutathione, oxidized cysteine, DROMs, superoxide, peroxynitrite, and NAD(P)H oxidase. Therefore, these studies have not established conclusively cause and effect nor have they provided mechanistic insight (Carnes et al., 2007; Neuman et al., 2007; Antoniades et al., 2012). Therefore, most of our knowledge about the possible mechanisms by which excess ROS can induce arrhythmia is from experimental and isolated cellular studies, which may not be applicable in clinical AF.

H_2_O_2_ prolongs the action potential duration (APD) and induces triggered activity (TA) via early afterdepolarization (EAD) and delayed afterdepolarization (DAD) mechanisms in myocytes (Beresewicz and Horackova, 1991). Perfusion of H_2_O_2_ (0.1–1 mM) into fibrotic rat and rabbit hearts in the Langendorff setting induces EADs, TAs, and subsequent arrhythmia (Morita et al., 2009). One of the mechanisms of H_2_O_2_-induced APD prolongation and EAD formation is by the development of an enhanced late sodium (Na^+^) current (Song et al., 2006). Treatment with H_2_O_2_ and angiotensin II enhance the late Na^+^ current but decreases the overall Na^+^ current in isolated myocytes through the down-regulation of SCN5A transcription (Shang et al., 2008). The antiarrhythmic effect of the late Na^+^ current blocker, ranolazine supports a role for the late Na^+^ current in mediating the genesis of EADs by oxidative stress (Morita et al., 2011). Nevertheless, one should consider that ranolazine has multiple other effects including its metabolic effects and blockade of Ikr current and that the role of late Na^+^ current in AF is not universally accepted (Schotten et al., 2010). While increase in late Na^+^ current may result in arrhythmia via an EAD mechanism, the reduction in total Na^+^ current caused by ROS may cause a reduction in CV and provide a substrate for reentry. We have shown that ROS can downregulate cardiac Na^+^ channels, and mitochondrial antioxidants can reverse this effect (Liu et al., 2010). ROS also can directly stimulate the L-type Ca^2+^ current, which results in abnormal intracellular calcium cycling in myocytes and facilitates EADs (Thomas et al., 1998). The studies on the net effect of ROS on L-type Ca^2+^ current have shown conflicting results, however. For example, L-type Ca^2+^ was found to be decreased in isolated atrial myocytes of patients with AF, probably via S-nitrosation of the calcium channels, and the current was restored to normal level by using N-acetylcysteine (Carnes et al., 2007). Hydroxyl radicals increase the open probability of cardiac ryanodine receptors, which control the Ca^2+^ release from the sarcoplasmic reticulum (SR) to the cytoplasm (Anzai et al., 1998). Excess ROS also increase I_to_ current probably via an increase in expression of the regulatory β-subunit KChlP2 (Sridhar et al., 2009).

A key factor in arrhythmogenesis is reduction of the repolarization reserve. This refers to the balance of inward depolarizing currents such as Na^+^ and Ca^2+^ and to the outward repolarizing currents such as potassium (K^+^) during the second and third phases of the cardiac action potential. Decreasing the repolarization reserve occurs when the balance is shifted away from repolarizing currents and results in a prolonged action potential and increased the likelihood of EADs and TA. The repolarization reserve and the cytoplasmic Ca^2+^ level of cardiac myocytes are affected by the rate of Ca^2+^ uptake by the SR. Exposure to OH^−^ significantly decreases SR Ca^2+^ uptake, which leads to an increased Ca^2+^ level in myocytes during diastole (Morris and Sulakhe, 1997). This short-term effect on Ca^2+^ transport is likely because of the OH^−^-mediated peroxidation of lipid membranes and protein sulfhydryl formation, which leads to an indirect effect on the SR Ca^2+^ transporter (Morris and Sulakhe, 1997).

ROS also affect gap junctions. Gap junctions form connections between cells through aggregation of connexin (Cx) proteins into hemichannels that meet to form conductive channels at cardiomyocyte interactions. Ventricular gap junctions are formed primarily from Cx43, however, a significant portion of gap junctions in the atria are formed by Cx40. c-Src is known to be activated by ROS. In an animal model of MI, the up-regulation of c-Src tyrosine kinase and an increase in the level of phosphorylated Tyr 416 c-Src (the active form of c-Src) resulted in the down-regulation of connexin43 (Cx43) via competition between phosphorylated c-Src and Cx43 for a binding site at zonula occludens-1, an intercalated disk scaffolding protein (Kieken et al., 2009). Other mechanisms for the reduction of Cx43 activity via the up-regulation of c-Src have also been suggested. They include tyrosine phosphorylation of Cx43 by c-Src, which also impairs gap junction function (Toyofuku et al., 1999). Inhibition of c-Src prevents Cx43 remodeling and ventricular arrhythmia caused by of angiotensin II activation and oxidative stress (Iravanian et al., 2011; Sovari et al., 2011b).

## ROS promotes other arrhythmogenic processes

AF is multifactorial. Inflammation, myocardial fibrosis, and oxidative stress are important pathologic events that promote AF (Negi et al., 2010; Sovari and Dudley, 2010). Activated inflammatory cells such as monocytes, neutrophils, eosinophils, and macrophages produce ROS and lysosomal hydrolytic enzymes at sites of inflammation (Morel et al., 1991). Also, ROS enhance the inflammatory response partially via the activation of signaling events that mediate the expression of inflammatory genes (Suzuki et al., 1997) in part through activation of nuclear factor-kappa B (NF-κB; Kabe et al., 2005). NF-κB, a family of related transcription factors that act as principal regulators of inflammation, are activated by various stimuli (such as ROS) after MI, in ischemic states, and during reperfusion (Lu et al., 2004; Seddon et al., 2007). We recently showed that the elevation of ROS levels by angiotensin II can activate NF-κB; this in turn transcriptionally downregulates Na^+^ currents (Shang et al., 2008). Nevertheless, whether the cardiac Na^+^ current is actually decreased in AF is controversial (Bosch et al., 1999; Sossalla et al., 2010; Schotten et al., 2011).

ROS enhance fibroblast proliferation and type I collagen gene expression (Murrell et al., 1990). Antioxidant therapy reduces fibrosis by decreasing the level of transforming growth factor-β, which is the major cytokine that promotes cardiac fibrosis (Koli et al., 2008; Cu et al., 2009). In addition, Ca^2+^/calmodulin-dependent protein kinases II (CaMKII) has been recently identified as one of the mediators of fibroblast proliferation in response to angiotensin II (Bellocci et al., 2007). Because CaMKII activity increases in the presence of oxidative stress, CaMKII activation may be a pathway by which oxidative stress stimulates fibroblast proliferation within the myocardium.

Therefore, excess amount of ROS can promote AF by effects on ion channels and by enhancing myocardial fibrosis. The level of excess ROS may vary in different conditions and may be a factor to determine the specific downstream effects of ROS; a matter that requires more studies.

## Oxidative stress and thromboembolism

Virchow's triad of blood stasis, endothelial dysfunction, and a hypercoagulable state are the main etiologic factors determining thrombus formation. The presence of left atrial spontaneous echo contrast and chamber enlargement, both of which are evidence of blood flow stasis in patients with AF, are strongly associated with an increased risk for cerebral ischemic events in this condition (Celermajer et al., 1994; Jones et al., 1996). The proclivity for thrombus formation in the LAA, emphasizes the central role of stasis in thromboembolism associated with AF. Oxidative stress can potentially contribute to the risk of thrombus formation in AF by causing endocardial dysfunction, and endocardial dysfunction has been demonstrated in AF. AF is associated with the downregulation of NO^•^ and the upregulation of superoxide production in the left atrium (Radomski et al., 1987), which shifts the endocardial balance toward thrombogenicity with overexpression of the prothombotic protein, plasminogen activator inhibitor-1, and increased expression of adhesion molecules on the endothelial surface (Bouchie et al., 1998; Carnes et al., 2001; Cai et al., 2002).

## Antioxidant therapies for atrial fibrillation

Antioxidant therapeutic agents for management of AF can be designed against at least three categories of targets: (1) ROS molecules, (2) the sources of ROS production, and (3) the key signaling molecules that mediate the arrhythmogenic effect of ROS (Sovari et al., 2011a; Figure 1).

One therapeutic approach for suppression of ROS is by using oxygen-radical scavengers such as vitamin E, vitamin C, N-acetyl-cysteine, ebselen, and tempol, which neutralize ROS molecules. Administration of vitamin C may reduce the incidence of postoperative AF (Carnes et al., 2001). Nevertheless, oxygen-radical scavengers have failed to show an impressive therapeutic effect for cardiovascular disorders in most clinical trials (Sesso et al., 2008; Song et al., 2009). ROS usually are highly reactive molecules, and conventional antioxidants may not be able to neutralize the ROS molecules before they exert their effect on proteins and lipids. In addition, ROS include a wide range of molecules, which may be generated from other ROS in reactions that are catalyzed by a wide range of enzymes. Table 5 reviews some of the known changes in important pro-oxidant or antioxidant enzymes in AF. Table 6 provides a review of some of the known downstream arrhythmogenic effects of only a few of the most important ROS molecules. A ROS scavenger may not effectively neutralize all ROS molecules to prevent the arrhythmogenic downstream effects. This may explain the lack of therapeutic effects of conventional antioxidants.

**Table 6:** **A review of some of the known arrhythmogenic molecular targets of ROS molecules**.

**ROS molecule**	**Known arrhythmogenic targets**
Superoxide	CaMKII (Kawakami and Okabe, 1998; Erickson et al., 2011), RyR (Kawakami and Okabe, 1998), L-type Ca^2+^ channels (Di Wang et al., 1999), SERCA (Tong et al., 2010), sodium channels (Tu et al., 2012), c-Src (Pu et al., 1996), NCX (Blaustein and Lederer, 1999)
Hydrogen peroxide	CaMKII (Erickson et al., 2011), RyR (Shan et al., 2010), L-type Ca^2+^ channels (Thomas et al., 1998), SERCA (Dremina et al., 2007), sodium channels (Ma et al., 2005), c-Src (Brumell et al., 1996; Yoshizumi et al., 2000), NCX (Soliman et al., 2009)
Hydroxyl radical	RyR (Anzai et al., 1998), SERCA (Morris and Sulakhe, 1997), L-type Ca^2+^ channels (Shirotani et al., 2001), NCX (Ziegelstein et al., 1992)
Peroxynitrite	RyR (Fauconnier et al., 2010), SERCA (Adachi et al., 2004), L-type Ca^2+^ channels (Mallet, 2005), sodium channels (Gautier et al., 2008), NCX (Chesnais et al., 1999)

CaMKII, Ca^2+^/calmodulin-dependent protein kinases II; NCX, Na^+^/Ca^2+^ exchanger; ROS, reactive oxygen species; RYR, ryanodine receptor; SERCA, sarco/endoplasmic reticulum Ca^2+^-ATPase.

A more effective therapeutic approach may involve inhibition of the sources of excess cardiac ROS. NAD(P)H oxidase activity has been shown to increase in AF (Dudley et al., 2005). The NAD(P)H oxidase is upregulated in early stages of AF but not in chronic AF (Reilly et al., 2011a). Therefore, inhibition of the NAD(P)H oxidase is most likely to be an effective treatment for primary prevention of AF and for postoperative AF (Sovari et al., 2008; Sovari, 2011). The association of NAD(P)H oxidase activity with development of postoperative AF supports the idea of using NAD(P)H oxidase inhibitors for prevention of AF following surgeries (Kim et al., 2008).

The effectiveness of NOS inhibitors such as *N*^G^-Nitro-L-arginine methyl ester (L-NAME) in treating arrhythmias has been tested in various experiments. In a model of occlusion-reperfusion arrhythmia in cats, it was shown that repeated injections of L-NAME decreased the incidence of occlusion arrhythmias by 40%, eliminated reperfusion-induced ventricular arrhythmias, and reduced the latency of occlusion arrhythmias (Kukushkina et al., 1999). More studies are required to evaluate the effect of NOS inhibitors on AF. One potential problem with NOS inhibition is the simultaneous reduction of NO with inhibition of ROS produced by uncoupled NOS. A more effective way to prevent ROS production by uncoupled NOS may be prevention of uncoupling by providing the required coenzyme, tetrahydrobiopterin. In a canine model of nonischemic heart failure with increased propensity to AF, treatment with tetrahydrobioptein and L-arginine prevented oxidative stress in the atrial tissue (Nishijima et al., 2011).

Mitochondrial ROS seems like an attractive therapeutic target. A large portion of cardiomyocytes are occupied by mitochondria, and they are major sources of cardiac ROS (O'Rourke et al., 2005). We tested the effect of seven different antioxidant therapies on prevention of ventricular arrhythmia in an angiotensin II activation mouse model with increased levels of ROS (Sovari et al., 2011c). ROS were highly compartmentalized in mitochondria and a mitochondria-targeted antioxidant prevented spontaneous and pacing induced ventricular arrhythmia. Whether this result can be applied to AF remains to be tested.

When an antioxidant agent that inhibits sources of ROS is designed, several important factors must be considered. Various sources of ROS can be activated under different pathologic conditions; therefore, an antioxidant against a specific source of ROS may be effective in the prevention of arrhythmia only under certain conditions. In addition, because of positive feedback loops among the sources of cardiac ROS, the simultaneous targeting of several important sources of ROS may prove to be an effective therapy.

A third therapeutic strategy may target signals that are downstream from ROS. For example, CaMKII inhibition may prevent many of the ROS-mediated effects on Ca^2+^ and Na^+^ channels or on the promotion of the fibrosis that causes arrhythmia. Ranolazine, a late Na^+^ current blocker, may inhibit some of the arrhythmogenic effects of ROS. We showed that the inhibition of c-Src tyrosine kinase prevents the effects of angiotensin II and ROS on Cx43 remodeling (Sovari et al., 2010, 2011b), and c-Src is activated by ROS (Sovari et al., 2011d). Thus c-Src may be another example of antiarrhythmic therapeutic targets that are probably downstream from oxidative stress.

In addition to the aforementioned three categories of antioxidant therapy, it is also possible to target the upstream pathologies that result in activation of sources of ROS. For example, angiotensin II is known to increase ROS production by activation of NAD(P)H oxidase and probably by increasing ROS production in mitochondria (Iravanian et al., 2008; Dikalov, 2011; Sovari et al., 2011c; Jeong et al., 2012). Angiotensin converting enzyme (ACE) inhibitors and angiotensin receptor blockers (ARBs) exert antioxidant and antiarrhythmic effect (Kober et al., 1995; Schramm et al., 2012). Nevertheless, there are numerous pathological insults that may activate sources of ROS such as ischemia-reperfusion, inflammation, aging, diabetes, tachycardia, mechanical stretch and sheer stress, and there are probably more unknown causes of oxidative stress (Molyneux et al., 2002; Boldt et al., 2003; De Champlain et al., 2004; Issac et al., 2007; Kregel and Zhang, 2007; Li et al., 2008; Van Wagoner, 2008; Ahmed et al., 2010; Morita et al., 2011; Sovari et al., 2012). Many of those pathological processes are complex, and no effective therapy is available for them. Moreover, some of the available therapies for these upstream targets are not completely effective. For example, ACE inhibitors and ARBs do not completely suppress the elevated levels of angiotensin II (Jorde et al., 2000; van de Wal et al., 2006).

An important consideration in the treatment of AF is early intervention. In later stages of chronic AF, the disease may be associated with multiple pathological processes, and some of the remodeling may not be reversible. For example, ACE inhibitors and ARBs are effective in primary prevention of AF; however, they may not be effective in the management of chronic AF (Disertori et al., 2009; Goette et al., 2012; Khatib et al., 2012). Similarly, antioxidant therapy may be much more effective if it is applied in early stages of AF and in primary prevention. Postoperative AF is an example for opportunities to test the efficacy of an early intervention. Statin drugs that exert some antioxidant activity by Rac1-mediated suppression of NAD(P)H oxidase have been shown to be effective in prevention of postoperative AF (Reilly et al., 2011b; Antoniades et al., 2012).

While chronic AF with significant structural and electrical remodeling of the heart is almost certainly a complex and multifactorial disease, the AF may be multifactorial from the onset. For example, the cause of a new onset AF in an elderly patient with diabetes and cardiomyopathy may be because of a combination of increased myocardial fibrosis, abnormal intracellular Ca^2+^ handling, autonomic dysfunction, and multiple abnormalities in ionic currents. Oxidative stress may be only one of the underlying pathologies. Therefore, the best antioxidant therapy may not completely prevent AF in all patients because, in some AF patients, other arrhythmogenic processes independent from oxidative stress exist.

Important guides in selecting those AF patients who will most likely benefit from antioxidant therapy are markers of oxidative stress. There are numerous serum markers of oxidative stress available such as thiobarbituric acid-reacting substances, superoxide dismutase and glutathione peroxidase activities (Hartnett et al., 2000), thioredoxin, ischemia-modified albumin (Lambrinoudaki et al., 2009), carotenoids, oxidized low density lipoproteins, oxidized low density lipoprotein antibodies (Suzuki et al., 2003), oxidized to reduced glutathione and cysteine, and DROMs (Neuman et al., 2007; Shimano et al., 2009). Some of these serum markers of oxidative stress have been shown to be elevated in AF patients; however, however, more studies are required to identify the best serum markers for patients at risk of AF and for those AF patients who possibly will respond to antioxidant treatment.

## Conclusions and future directions

Treatments that do not address the underlying pathophysiology of AF are likely to have limits to their efficacy. There is considerable evidence that oxidative stress has an important role in genesis of AF. The pathological link remains to be proven, however, and designing an effective antioxidant therapy requires a better understanding of the complex biology of oxidative stress.

Current evidence suggests that NAD(P)H oxidase inhibitors may be effective in primary prevention of AF and in postoperative AF. Mitochondria-targeted antioxidants may prove to be most effective antioxidant therapeutic intervention in persistent AF. Targeting important molecules downstream from ROS such as c-Src and CaMKII may provide additional antiarrhythmic effect. Effective antioxidant therapy may also reduce the risk of thromboembolism in patients with AF by improving endocardial dysfunction.

### Conflict of interest statement

Dr. Dudley has applied for patents: (1) Method for Predicting Onset/Risk of Atrial Fibrillation, (2) Prevention of sudden death by modulation of Src family, (3) Modulating mitochondrial reactive oxygen species to increase cardiac sodium channel current and mitigate sudden death, (4) Biomarkers for Prediction of Stroke Risk in Atrial Fibrillation, and (5) Mitochondrial anti-oxidants for prevention of sudden death by raising connexin43 levels.
